# Keap1 hypomorphism protects against ischemic and obstructive kidney disease

**DOI:** 10.1038/srep36185

**Published:** 2016-11-02

**Authors:** Roderick J. Tan, Dionysios V. Chartoumpekis, Brittney M. Rush, Dong Zhou, Haiyan Fu, Thomas W. Kensler, Youhua Liu

**Affiliations:** 1 Department of Medicine, University of Pittsburgh School of Medicine, Pittsburgh, Pennsylvania, USA; 2 Department of Pharmacology and Chemical Biology, University of Pittsburgh School of Medicine, Pittsburgh, Pennsylvania, USA; 3 Department of Pathology, University of Pittsburgh School of Medicine, Pittsburgh, Pennsylvania, USA.

## Abstract

The Keap1/Nrf2 pathway is a master regulator of antioxidant, anti-inflammatory, and other cytoprotective mechanisms important in protection from kidney disease. For the first time in kidney disease, we describe the use of Keap1 hypomorphic mice, which possess Nrf2 hyperactivation. We exposed these mice and wild type controls to ischemia/reperfusion injury (IRI). The initial tubular injury at 24 hours post-IRI appeared to be unaffected, with the only observed difference being a decrease in inflammatory cytokine expression in the hypomorphs. However, we noted significant improvement in serum creatinine in the hypomorphs at 3 and 10 days after injury, and renal fibrosis was dramatically attenuated at the late timepoint. Assessment of Nrf2-regulated targets (GSTM1, GSTP1, NQO1) revealed higher expression in the hypomorphs at baseline. While injury tended to suppress these genes in wild-type mice, the suppression was attenuated or reversed in Keap1 hypomorphs, suggesting that protection in these mice was mediated by increased Nrf2 transcriptional activity. To assess the generalizability of our findings, we subjected the hypomorphs to unilateral ureteral obstruction (UUO) and again found significant protection and increased expression of Nrf2 targets. Overall, these results support the conclusion that the Nrf2 pathway is protective in a variety of kidney diseases.

Treatment of kidney disease continues to be a significant challenge for modern medicine. Outside of supportive care, there are no proven therapies to promote renal recovery after acute kidney injury (AKI) in humans^1^. Furthermore, chronic kidney disease (CKD) treatment has relied solely upon blockade of the renin-angiotensin system for more than two decades^2^. The afforded protection is neither complete nor without complications^3^,^4^. New therapies are required if we are to effectively improve prognosis of renal disease in patients.

The Keap1/Nrf2 pathway is a master regulator of cytoprotective genes and a promising target for therapeutic intervention in kidney disease. Nrf2 (nuclear factor erythroid 2 like 2) is a transcription factor with the ability to bind antioxidant response elements (ARE) in target gene regulatory regions. Upregulated genes are involved in antioxidant defense as well as conjugation/detoxification reactions for drugs and xenobiotics^5^. An anti-inflammatory role of Nrf2 via interaction with the nuclear factor-κB pathway has also been described^6^.

Keap1 (Kelch-like ECH associated protein-1) is a cytoplasmic inhibitor of Nrf2 and an adaptor for the Cullin 3 ubiquitin ligase. When bound to Keap1, Nrf2 is targeted for polyubiquitination and degradation by the proteasome. Disruption of the Keap1-Nrf2 complex allows for Nrf2 nuclear accumulation with subsequent modulation of gene transcription^5^.

Keap1 inactivation is mediated by cysteine residues in this protein that, when modified by oxidative or electrophilic stress, cause a conformational change that impedes the polyubiquitination and subsequent degradation of Nrf2^5^. Keap1 is therefore an intracellular sensor for cellular stress and its inhibition or genetic disruption leads to increased Nrf2 pathway activation. A number of pharmacological agents, such as CDDO-Me (bardoxolone methyl), CDDO-Im, and sulforaphane have all been shown to enhance Nrf2 pathway activation by modifying the reactive cysteines of Keap1^6^,^7^,^8^.

The Keap1/Nrf2 pathway has been previously implicated in renal injury. In ischemia/reperfusion injury (IRI), it was found that Nrf2 signaling is upregulated in response to injury, and that Nrf2 null mice developed worse disease^9^. It was also found that bardoxolone methyl, CDDO-Im, and sulforaphane could ameliorate IRI, if given before injury initiation^8^,^10^,^11^. In CKD models, it was found that Nrf2 enhancers could decrease diabetic and aristolochic acid nephropathy^12^,^13^. However, these prior studies did not assess AKI-to-CKD progression. Furthermore, these studies relied upon pharmacologic enhancement of the Nrf2 pathway which may have unintended off-target effects.

To examine the role of enhanced Nrf2 activity in AKI and CKD, we chose to use genetically modified mice to avoid potentially confounding off-target effects. Unfortunately, Keap1 null mice are nonviable due to esophageal obstruction^14^. We turned instead to Keap1 hypomorphs, which were originally generated via the introduction of the floxed allele of Keap1 as an attempt to generate tissue specific knockouts. However, it was fortuitously discovered that these floxed alleles led to underproduction of Keap1 (hypomorphism) and resulted in elevated Nrf2 transcriptional activity^15^.

In designing our experiments, we were careful to avoid renal injuries related to chemical agent exposures. The Keap1/Nrf2 pathway is a regulator of cellular detoxifying mechanisms—two of the genes assessed in this study, *GSTM1* and *GSTP1*, are key components of this process. Use of chemical agents such as adriamycin would therefore be confounded by the effects of Keap1 hypomorphism on chemical metabolism, possibly leading to altered renal exposure to the drug. To circumvent this issue, we focused on surgical models of kidney disease.

We therefore exposed these Keap1 hypomorphic mice to renal injury by IRI. While little histologic or functional difference was noted 24 hours after injury, there was a reduction in proinflammatory cytokine production in the hypomorphs. The hypomorphs eventually demonstrated functional protection at 3 days and very dramatically at 10 days after injury. The protection was associated with improvements in histology, biochemical markers, and Nrf2 target gene expression levels. Similar findings were demonstrated in the unilateral ureteral obstruction (UUO) model. These data suggest that the Nrf2 pathway can be protective in a variety of renal diseases and is a viable target for renoprotective interventions.

## Results

### Keap1 hypomorphs are minimally protected in the early stage of AKI

**Figure 1:**
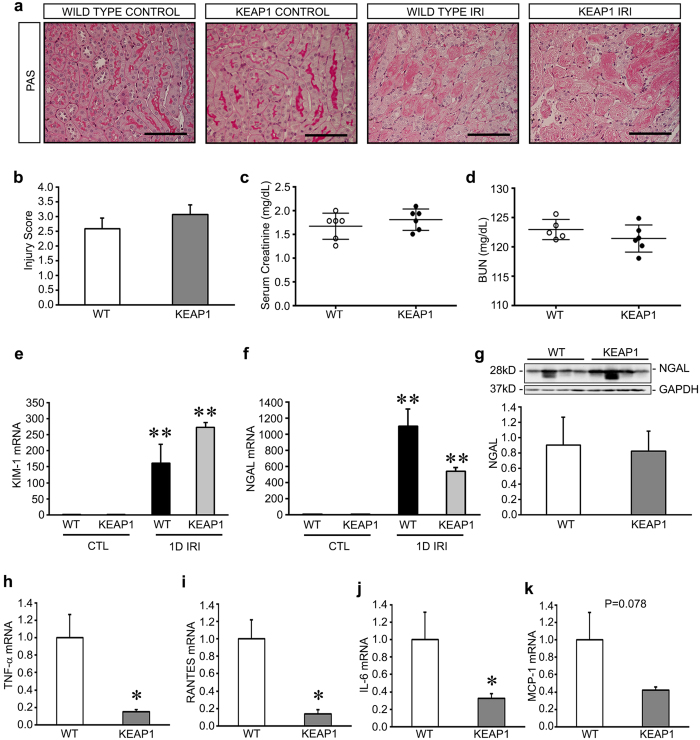
Keap1 hypomorphs demonstrate minimal protection 24 hours after ischemia-reperfusion injury (IRI). Keap1 hypomorphs (KEAP1) and wild type (WT) controls were subjected to unilateral renal IRI with simultaneous contralateral nephrectomy. (**a**) Histological assessment with Periodic Acid Schiff (PAS) staining showed similar tubular damage after injury, as manifested by tubular necrosis and loss of brush borders, in both sets of mice compared to the untreated control kidney (which themselves were not different between groups). Bar equals 100 μm. (**b**) Quantitative analysis confirmed no detectable difference in histologic injury (n = 6 for each group). (**c,d**) There were no significant differences in serum creatinine and BUN between groups. Each dot represents an individual animal with the mean ± SEM superimposed. (**e,f)** After IRI, qRT-PCR analysis of tubular injury markers KIM-1 and NGAL revealed significant increases from the control kidneys, but no difference between WT and KEAP mice. (**P < *0.05* compared to either CTL untreated kidney values, one-way ANOVA, n = 4). (**g**) Western blot analysis and densitometry of NGAL confirms no significant difference between injured WT and KEAP1 kidneys. NGAL was not detectable by this method in uninjured kidneys. (**h–k**) Proinflammatory mediators were reduced in KEAP1 compared to WT mice (n = 5–6 in each group). (*P < *0.05* compared to WT mice, *P* = *0.078* for MCP-1.)

To test the effect of Keap1 depletion on kidney injury, we exposed Keap1 hypomorphs to unilateral IRI. The generation and characterization of these mice were previously described^15^. At baseline, there were no significant differences between Keap1 hypomorph levels of serum creatinine and blood urea nitrogen (BUN) levels compared to wild type controls (Supplementary Fig. 1a,b). To induce injury, unilateral ischemia was performed via clamping of the left renal pedicle for 35 minutes. The contralateral kidney was removed in the same surgery and used as an untreated internal control, and were histologically indistinguishable between hypomorph and wild type groups (Fig. 1a). Mice were sacrificed 24 hours after reperfusion. All IRI kidneys exhibited significant histologic injury, with loss of brush borders, coagulation necrosis, and tubular damage, and there was no significant difference between wild type and hypomorphic mice (Fig. 1a,b). Similarly, there was no difference in kidney function as measured by serum creatinine and blood urea nitrogen (BUN) (Fig. 1c,d). Tubular injury markers such as *Kidney Injury Molecule-1 (KIM-1)* and *Neutrophil Gelatinase Associated Lipocalin (NGAL)* were also not significantly different by mRNA expression in either group, although the expression of these markers was elevated in both groups after injury (Fig. 1e,f). Further, although we were unable to detect NGAL by western blot in nonischemic kidneys, we were easily able to detect it in IRI kidneys, and we confirmed there was no significant difference between the wild-type and hypomorph groups (Fig. 1g).

However, we were able to discern dramatic reductions in the expression of inflammatory mediators *tumor necrosis factor-a* (*TNF-α*)*, RANTES, interleukin-6* (*IL-6*) and *monocyte chemotactic protein-1* (*MCP-1*) (Fig. 1h–k) in mutant mice. There were no significant differences in inflammatory cytokines in the untreated, nonischemic kidneys at baseline (Supplementary Fig. 1c–e). This strongly suggests that despite a lack of early effects on tubular histology and function, as well as the release of tubular injury markers, there is an underlying anti-inflammatory effect in hypomorphs that could play a role in disease progression.

### Keap1 hypomorphs have decreased injury in later stages of IRI

Considering the differences in inflammatory mediators at the 24 hour timepoint, we examined later timepoints after IRI to assess the evolution of this injury. We continued to use unilateral ischemia, this time with a contralateral nephrectomy performed exactly 24 hours prior to sacrifice to allow for the use of serum markers to assess injury. The unilateral IRI model has the advantage of being very reproducible, and the presence of the normal contralateral kidney ensures that all animals survive to late timepoints (as bilateral IRI is associated with significant mortality), as has been previously described^16^.

**Figure 2:**
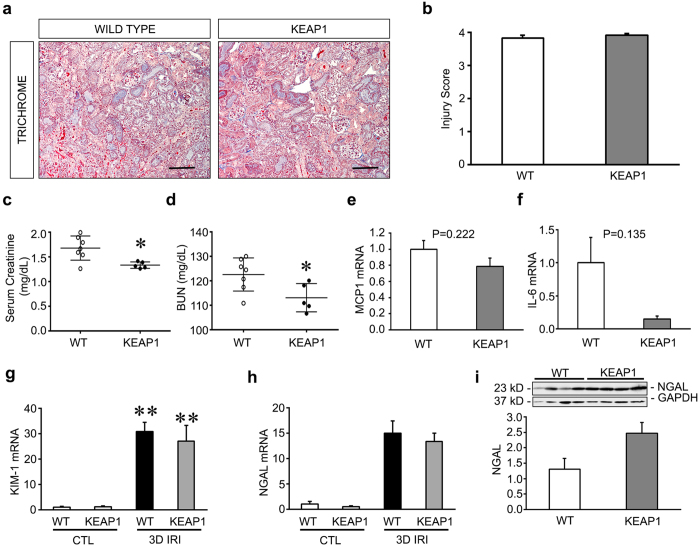
Keap1 hypomorphs have improved renal function 3 days after ischemia-reperfusion injury (IRI). Wild type (WT) and hypomorph mice (KEAP1) were subjected to unilateral renal IRI, with a contralateral nephrectomy performed 24 hours prior to sacrifice at 3 days. (**a,b**) Histologic assessment of kidneys showed significant tubular injury with no perceptible difference between groups. Bar equals 100 μm. (**c,d**) Serum creatinine and BUN were significantly improved in the hypomorphs in spite of the lack of histologic differences. Each dot represents an individual animal with mean ± SEM shown. (**e,f**) qRT-PCR showed no significant reductions in proinflammatory mediators (n = 4–5 in each group). (**g,h**) qRT-PCR analysis of tubular injury markers KIM-1 and NGAL showed a significant increase (KIM-1) or trend to increase (NGAL) in injured kidneys vs CTL uninjured kidneys, but no significant difference between injured WT and KEAP1 kidneys. (**j**) Western blot and densitometry for NGAL confirms no decrease in NGAL in injured KEAP1 kidneys compared to injured WT kidneys. (*P < *0.05* compared to the wild type group. **P < *0.05* compared to either CTL group).

Three days after reperfusion we again found no perceptible changes in histologic injury (Fig. 2a,b). However, at this timepoint serum creatinine and BUN levels were significantly improved in the Keap1 hypomorphs (Fig. 2c,d). The previously noted differences in inflammatory mediators were not present (*MCP-1* in Fig. 2e, *TNF-α* and *RANTES* not shown), except for a nonsignificant decrease in *IL-6* mRNA (Fig. 2f, *P* = *0.135*). This may suggest that an initial wave of inflammation has resolved by this timepoint. Consistent with the histologic findings and results of the 24 hour IRI experiment, we again could not detect any difference between the two groups of mice in *KIM-1* and *NGAL* mRNA expression or NGAL protein levels, even though both markers increased dramatically after injury in both groups (Fig. 2g–i). This at least suggests that the tubular injury itself was equally severe in both groups of mice.

**Figure 3:**
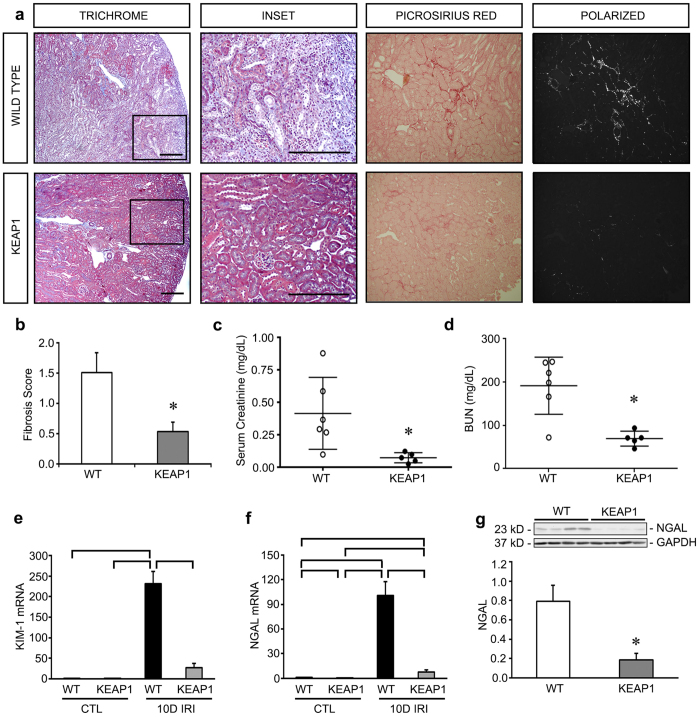
Keap1 hypomorphs demonstrated unequivocal protection 10 days after ischemia-reperfusion injury (IRI). Keap1 hypomorphs (KEAP1) and wild type (WT) mice were subjected to unilateral renal IRI, with a contralateral nephrectomy performed 24 hours prior to sacrifice at 10 days. (**a**) Kidney sections were subjected to Masson’s Trichrome staining to evaluate for fibrosis development (collagen appears blue). WT mice also had more inflammatory cells. Low powered views are shown along with an enlarged inset of the boxed area. Bar equals 100 μm. Picrosirius red was also performed – under light microscopy collagen and other cellular components stain red. With polarized light of the same sections shown on light microscopy, birefringence is highly specific for collagen. (**b**) Keap1 hypomorphs had significantly decreased fibrosis, which was confirmed with fibrosis scoring (n = 5–6 for each group). (**c,d**) Serum creatinine and BUN were significantly reduced in the hypomorphs. Each dot represents an individual mouse with the mean ± SEM superimposed. (**e,f**) qRT-PCR for KIM-1 and NGAL shows significant reduction in these tubular injury markers in IRI KEAP1 kidneys compared to IRI WT kidneys. Brackets show significant differences, *P* < *0.05*. (**g**) NGAL was significantly suppressed in the IRI KEAP1 kidneys compared to IRI WT kidneys, confirming the qRT-PCR result in (**f**). (*P* < *0.05,* compared to similarly treated WT group).

**Figure 4:**
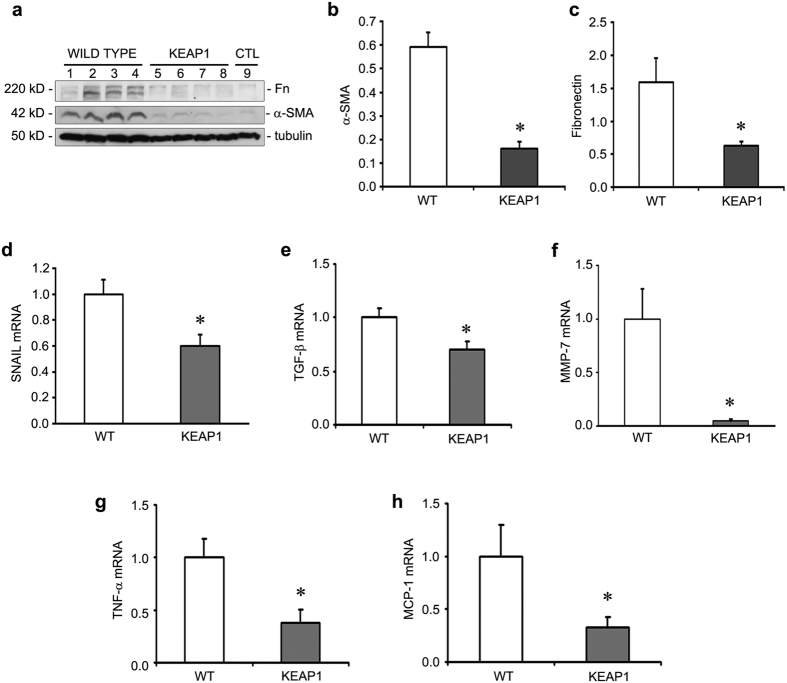
Keap1 hypomorphs were protected from the fibrotic and inflammatory response at 10 days after ischemia-reperfusion injury (IRI). (**a**) Western blot for fibrosis markers fibronectin (Fn) and smooth muscle actin (α-SMA) along with the loading control tubulin is shown for IRI wild type and KEAP1 mice, along with an untreated control (CTL) for reference. (**b,c**) Western blot densitometry of Fn and α-SMA (normalized to tubulin) shows significant reduction in the hypomorphs. (**d–f**) qRT-PCR of fibrosis-related genes *Snail, MMP-7*, and *TGF-β* (and *α-SMA*, data not shown) were significantly reduced in Keap1 hypomorphs. (n = 5–6 for each group) (**g,h**) Hypomorphs were also protected from increases in mRNA for proinflammatory cytokines *TNF-α* and *MCP-1*. (*P < *0.05* compared to the wild type mice. N = 5–6).

Unilateral IRI is characterized by the development of significant fibrosis at late stages after injury and is a useful model for evaluating AKI-to-CKD progression. It has been previously shown that renal fibrosis, a marker of CKD, is established as early as 10 days after AKI injury in mice^17^. In our experiments with Keap1 hypomorphs at 10 days post-AKI injury, we found dramatic differences in both histology and function. While wild type mice exhibited significant fibrosis demonstrated by both trichrome and picrosirius red stains, Keap1 hypomorphs were nearly completely protected from fibrosis (Fig. 3a,b). Accordingly, serum creatinine and BUN were also significantly improved in the hypomorphs (Fig. 3c,d). In contrast to the earlier timepoints, there was also a significant decrease in tubular injury markers in the IRI hypomorph kidneys compared to the similarly-treated wild type kidneys, suggesting true protection against tubular injury at 10 days post-IRI (Fig. 3e–g). These mice were also protected from increases in fibronectin, α-smooth muscle actin (α-SMA), *Snail*, *matrix metalloproteinase-7* (*MMP-7*), and *TGF-β* (Fig. 4a–f). These are all known mediators of renal fibrosis^18^,^19^. Inflammatory mediators were also reduced, including *TNF-α* and *MCP-1* (Fig. 4g,h), which may represent protection from the inflammation that is typically associated with development of fibrosis.

### Keap1 hypomorphs are protected from injury-induced suppression of Nrf2 activity

To validate the phenotype of the Keap1 hypomorphs we undertook experiments evaluating *Keap1* and *Nrf2* expression, as well as the target genes of Nrf2. These genes include members of the glutathione-S-transferase family (*GSTM1* and *GSTP1*) and NAD(P)H: quinone oxidoreductase 1 (*NQO1*), which play roles in detoxification and in preventing oxidative stress^20^,^21^,^22^.

**Figure 5:**
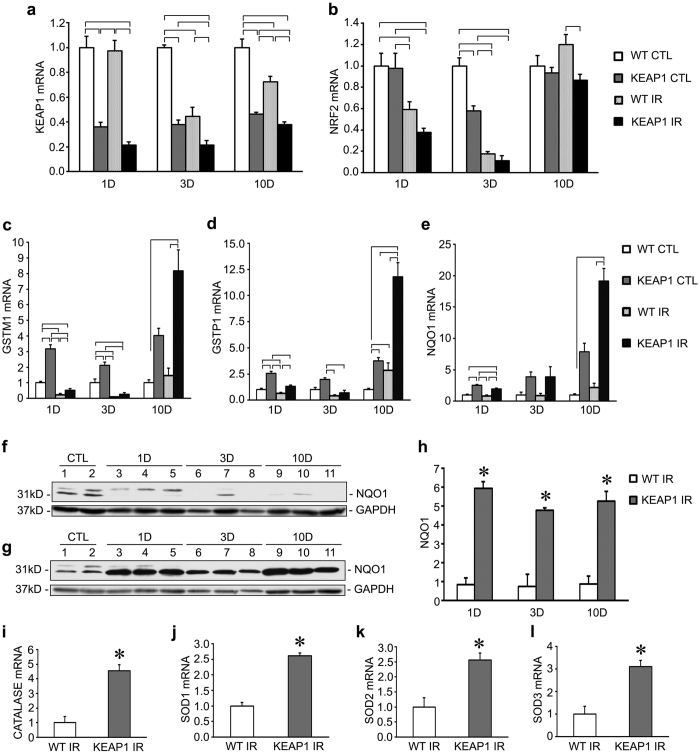
Nrf2 target genes are downregulated after injury but this is improved in Keap1 hypomorphs. We assessed Keap1, Nrf2, and Nrf2 targets in the IRI mice used for this study. (**a**) As expected, mRNA levels of Keap1 were consistently decreased at baseline in CTL kidneys in the hypomorphic mice compared to WT mice at all timepoints. At 3 and 10 days post-IRI, there was a decrease in Keap1 expression in all injured kidneys but the hypomorphs were consistently lower than the WT kidneys. Brackets indicate significant differences. (n = 4) (**b**) Nrf2 mRNA generally showed significant differences between injured and uninjured kidneys, but generally did not show significant differences between similarly treated WT and KEAP kidneys. (n = 4) (**c–e**) Gene expression levels of *GSTM1, GSTP1,* and *NQO1* were assessed. Brackets indicate groups with significant differences (n = 4–6 for each group). Comparing the uninjured control kidneys (CTL), Keap1 hypomorphs (KEAP1) had higher expression levels compared to wild type (WT) mice. Injury led to suppression of gene expression in all animals. However, this reduction was blunted in the hypomorphs and in general these mice had levels of gene expression that were either closer to, or much higher than, the WT CTL group. Western blots for NQO1 were performed for the wild type mice (**f**) and hypomorphs (**g**) over the course of IRI injury. Identical samples (WT CTL) were loaded into lanes 1 and 2 of both gels to facilitate comparison of all the samples over two gels. Densitometry of these bands (n = 3) normalized to GAPDH is shown in (**h**) and these values were in turn normalized to the samples in lanes 1 and 2. Additional genes were assessed in the 10 day IRI kidneys and revealed increased expression of protective antioxidant genes catalase and the three SODs (**i–l**). (*P < *0.05* compared to WT group. n = 5–6).

As expected for this mouse strain, the uninjured kidneys of the Keap1 hypomorphs had significantly lower expression of *Keap1* mRNA when compared to uninjured wild-type mice at all timepoints examined (Fig. 5a). This is in agreement with previous studies of this strain^15^. Interestingly, *Keap1* expression decreased in wild-type IRI kidneys at later timepoints, suggesting an attempt to upregulate Nrf2 activity. However, the hypomorphs generally had lower *Keap1* expression than the wild type mice regardless of treatment. On the other hand, *Nrf2* gene expression was not significantly affected (Fig. 5b), but this does not preclude effects on Nrf2 activity, since the effect of Keap1 on Nrf2 is post-translational as Keap1 directs Nrf2 for proteasomal degradation. There would not necessarily be any effect on *Nrf2* gene expression because of this. Unfortunately, although we wanted to assess Nrf2 protein abundance by western blotting, attempts with two different antibodies against Nrf2 were unsuccessful.

As such, we evaluated the expression of Nrf2 target genes. Comparing uninjured kidneys from both groups revealed significantly elevated Nrf2 targets at baseline in the hypomorphic mice. After injury, the wild type mice tended to have suppressed expression of target genes at the 1 and 3 day timepoints, which is likely maladaptive in the setting of injury and may promote disease progression. Although the Keap1 hypomorphs also demonstrated some degree of decreased expression of target genes after injury, the overall expression of these genes were still higher compared to the corresponding injured wild type kidneys. Furthermore, injured hypomorph kidneys often had gene expression levels that approximated or superseded the baseline levels of the uninjured wild type mouse kidneys (Fig. 5c–e).

Since NQO1 demonstrated the greatest fold increase after injury (Fig. 5e), we examined NQO1 protein levels via Western blot analysis. In comparison to untreated controls, the wild type mice had lower levels of NQO1 protein at all timepoints after IRI injury (Fig. 5f). Meanwhile the hypomorph mice had increased levels of NQO1 at all timepoints (Fig. 5g,h).

In the 10 day IRI kidneys, we examined an expanded set of potential Nrf2 targets, including catalase and all three superoxide dismutase isoforms^23^,^24^. Gene expression for these antioxidant enzymes were all significantly upregulated in the hypomorphs compared to the wild type mice (Fig. 5i–l). Of these genes, only catalase was upregulated in Keap1 mice at baseline or at day 1 and 3 after IRI, suggesting greater protection at 10 days after IRI compared to these earlier timepoints (Supplementary Fig. S2).

**Figure 6:**
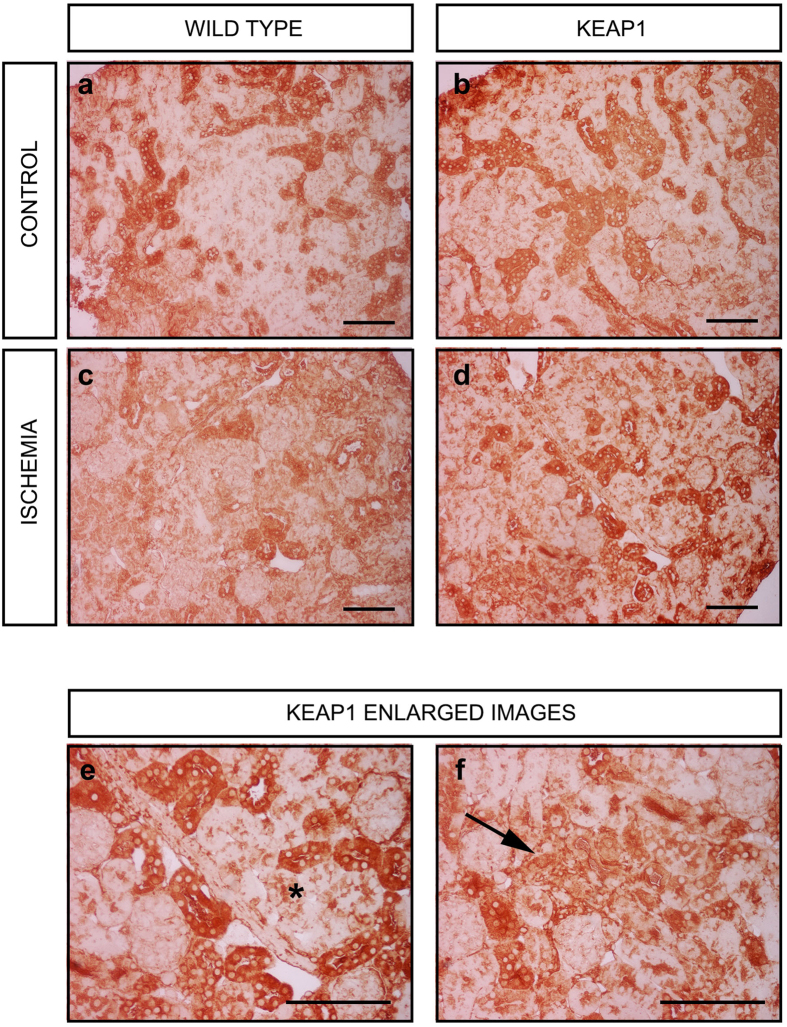
NQO1 expression is enhanced in renal tubules of Keap1 hypomorph mice. (**a,b**) Immunohistochemical staining reveals strong expression in a subset of renal tubules in control uninjured kidneys in both wild type and Keap1 hypomorphic mice. (**c**) There was loss of staining from renal tubules in injured wild type mice. (**d**) In hypomorphic mice staining was largely preserved. (**e,f**) Enlargement of areas of (**d**) show an enhancement in renal tubules (**e**) in the Keap1 hypomorphs (asterisk) as well as in a few rare interstitial areas (**f**) suggesting fibroblast expression. Bar equals 100 μm.

To determine what cell types were responsible for these changes, we undertook immunohistochemical staining for NQO1. NQO1 was essentially only expressed in a subset of renal tubules at baseline in uninjured control kidneys (Fig. 6a,b). However, after ischemia in the wild type mice, there was significant loss of NQO1 staining throughout the kidney (Fig. 6c). In the Keap1 hypomorphs there was maintenance of the NQO1 staining primarily in the renal tubules (Fig. 6d). In addition, there was increased staining for NQO1 in renal tubules that were not previously expressing this protein, giving a more homogenous staining appearance to the tissue (asterisk, Fig. 6e). In a few rare cases, interstitial cells upregulated NQO1 (arrow, Fig. 6f).

Collectively, these data first demonstrate that protective Nrf2 activities are suppressed after injury, a state which is likely maladaptive and may lead to further renal injury. Second, Keap1 hypomorphs are largely protected from this effect, which may explain their improved status after injury.

### Keap1 hypomorphs are protected from UUO injury

**Figure 7:**
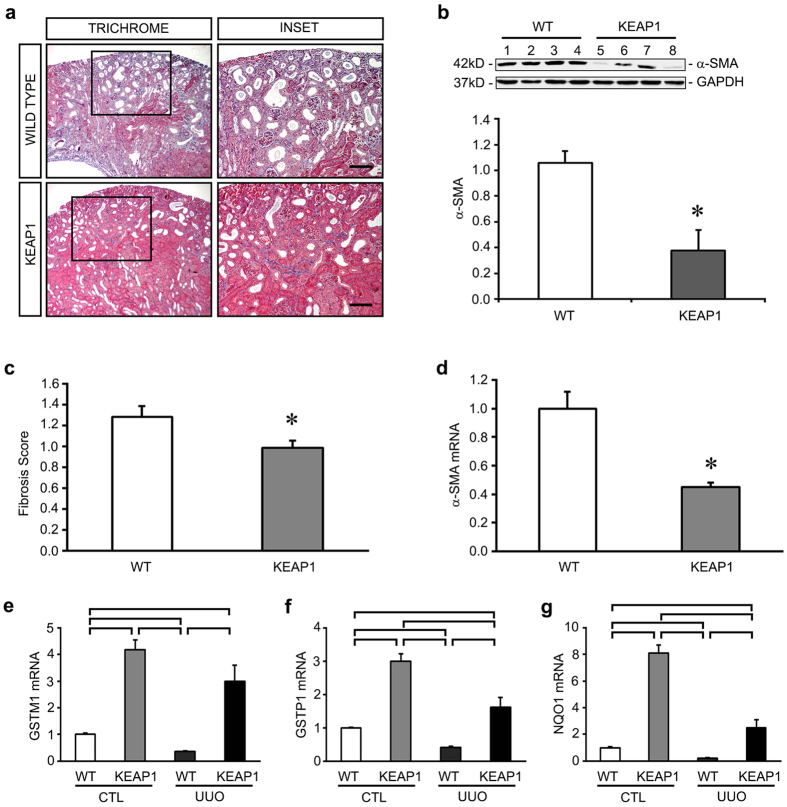
Keap1 hypomorphs (KEAP1) are protected from unilateral ureteral obstruction (UUO) injury. Both wild type (WT) and hypomorph mice (KEAP1) were subjected to UUO injury. (**a**) Trichrome stains reveal increased fibrosis (blue staining) in the wild type mice. (**b**) Western blot for *α-SMA* showed reduced expression in the hypomorphs. (**c**) Blinded scoring confirmed reduced histologic injury. (**d**) qRT-PCR for α-SMA showed reduced expression in hypomorphs. (**e–g**) Gene expression analysis of the Nrf2 target genes *GSTM1, GSTP1,* and *NQO1* revealed increased levels in the untreated contralateral kidneys (CTL) of the hypomorphs (n = 5 for each group). UUO injury led to suppression of these genes, but the hypomorphs were protected from this decline. Brackets show groups that are significantly different. (*P < *0.05* compared to WT group at the same timepoint).

To determine if the protective effect is generalizable to other kidney injuries, we subjected the Keap1 hypomorphs to injury via UUO. We found that hypomorphs were significantly protected from histologic injury 7 days after UUO (Fig. 7a,c). In addition, the expression of the fibrosis marker α-SMA was decreased in these mice (Fig. 7b,d). As in IRI, we assessed levels of Nrf2 target genes, finding that there was suppression of *GSTM1, GSTP1,* and *NQO1* after injury in both sets of mice. However, this suppression was blunted in the Keap1 mutants, and in the case of all three genes, expression levels in hypomorphs were at least equivalent to, and often greater than, wild type uninjured kidneys (Fig. 7e–g).

## Discussion

The Keap1/Nrf2 pathway has been implicated in a number of experimental renal diseases including ischemia/reperfusion injury^8^,^9^. However, these initial studies did not examine late timepoints after AKI/IRI, which represents progression to CKD^17^. Furthermore, these studies relied upon pharmacological enhancement of the Nrf2 pathway in order to demonstrate protection from disease.

The use of pharmacologic enhancers can have unintended negative consequences. For instance, in one study authors utilized RTA 405, an analog of bardoxolone methyl, in diabetic nephropathy in rats. However, instead of protection, the investigators actually found worsening of the renal disease as measured by proteinuria and histologic injury. During this study, it was discovered that the two lots of RTA 405 used to treat the rats had numerous impurities and degradation products that were not previously identified. Thinking that this was the cause of the unexpected outcomes, they obtained a different analog dh404 from the manufacturers. Again, dh404 actually worsened diabetic renal disease and was associated with the development of new tumor-like growths in the kidney^25^. Other experimental studies also showed potential for deleterious renal effects with this agent^26^,^27^.

Unfortunately, adverse events have also been described in human clinical trials. Initial results from a phase 2 trial using bardoxolone methyl in diabetic nephropathy revealed promising effects on estimated GFR at 24 weeks and one year after initiation^28^. However, this result was accompanied by concerns regarding hypomagnesemia and increases in proteinuria, the latter leading some investigators to suggest that the effects were mediated by altered glomerular hemodynamics rather than an intrinsic improvement in renal pathology^29^,^30^,^31^,^32^. The followup phase 3 trial in a larger population of patients with more advanced CKD was terminated early because of increased cardiovascular events in patients receiving the drug^33^. It is currently unclear whether the adverse effects are due to off-target actions of the drug or direct effects on the Nrf2 pathway itself.

In order to clarify contributions of the Nrf2 pathway, we utilized genetically altered mice that express low levels of the Keap1 protein (Fig. 5a). Reduction in levels of this inhibitor of Nrf2 signaling leads to enhanced Nrf2 target gene transcription (Fig. 5c–e). By excluding pharmacologic enhancers in our study we avoided any negative, and possibly even positive, off-target effects of these agents to obtain a more complete understanding of this pathway in renal disease.

In our hands, we determined that Keap1 hypomorphs gained protection from both IRI and UUO injuries. In IRI, there was no overt biochemical, functional or morphological protection of the renal tubules at the very early timepoint of 24 hours, whereas serum creatinine and BUN were improved at 3 and 10 days after injury (Figs 1, 2 and 3). The tubular injury markers KIM-1 and NGAL were only convincingly and consistently decreased in the hypomorph mice at 10 days. This suggests that the Keap1 hypomorphs were unable to protect renal tubules in the initial wave of injury, in spite of higher than normal expression of Nrf2 target genes (Fig. 5c–e). However as the injury progressed, the protective effect of Nrf2 became apparent. It has been noted that there are different and temporally separated waves of cell death after IRI injury, which could explain this phenomenon^34^.

Interestingly, although there was no overt difference between tubular injury at 24 hours after IRI between the hypomorph and wild type mice, there was a profound difference in expression of inflammatory mediators. The cytokine differences disappeared at the 3 day timepoint but became significant again at the late 10 day timepoint, providing evidence of an early, intermediate and late stage of inflammation which is affected in divergent ways by Keap1 hypomorphism.

Inflammation plays a critical role in the development of AKI. It has been proposed that acute inflammation may set the stage for a consolidation phase that determines the long-term outcome of the kidney^35^. Our findings of decreased inflammatory cytokine expression in Keap1 hypomorphs suggests that early suppression of inflammation can indeed lead to long-term beneficial effects on overall recovery from AKI, even when there is apparent equivalency of initial tubular damage. This is also consistent with earlier findings that AKI leads to long-term population of the kidney with lymphocyte subsets, and with findings that inhibiting inflammation leads to AKI protection^36^,^37^.

Our examination of the late fibrotic stage after AKI is a unique aspect of our study compared to previous studies and shows that Nrf2 can prevent disease progression and the development of CKD after AKI. At late stages of disease, the hypomorphs were protected from fibrosis as well as the upregulation of pro-fibrotic mediators, while maintaining expression of protective antioxidants (Figs 4 and 5). Similar findings were obtained in UUO injury (Fig. 7), another CKD model which has never before been examined in the Keap1 hypomorphs. In addition, we noted that the hypomorphs had lower gene expression of *Snail* and *MMP-7*. These are known targets of the Wnt/β-catenin signaling pathway, which has been strongly associated with AKI-to-CKD progression and fibrosis^38^,^39^. Potential interactions between Wnt/β-catenin and Nrf2 warrants further study.

As expected, Keap1 hypomorphs expressed lower levels of *Keap1* than the wild type mice regardless of treatment group (Fig. 5a). There was no consistent effect on *Nrf2* expression, but this is not unexpected as the effect of Keap1 is on post-translational Nrf2 regulation via proteasomal degradation. However, the renal protection of the Keap1 hypomorphs was associated with increased expression of Nrf2 target genes (Fig. 5c–e). This increase over wild type mice was noted in both uninjured and injured kidneys. Of interest, it appears that both IRI and UUO leads to a downregulation of Nrf2 target genes in wild type mice. Downregulation of cytoprotective genes is counterintuitive in the setting of renal injury, and may be at least one mechanism by which injury proceeds in either type of kidney injury. The maintenance of basal levels of these genes after injury may lead to protection, as gene expression of injured Keap1 hypomorph kidneys approximated levels observed in uninjured wild type kidneys. We acknowledge that other investigators have found an upregulation of Nrf2 target genes after IRI^11^,^21^. Our results may differ because of the differences in our experimental model, including total ischemia time and the particular mouse strains utilized which can have significant effects on the overall outcome^16^.

The specific Nrf2 targets evaluated were *GSTM1*, *GSTP1*, and *NQO1* which are important in detoxification of chemicals and xenobiotics as well as in antioxidant defense. Members of the GST family were previously shown to be altered in IRI injury^21^. The ability to resist chemical injury would be helpful for cellular survival in stressful disease situations. It is also not surprising that enhancement of antioxidants such as NQO1, catalase, and the SODs are protective in renal disease. We recently described a protective role for SOD3 during kidney injury as well as a loss of this antioxidant from the kidney during disease states^40^. We further discovered that NQO1 was largely localized to a subset of renal tubules prior to injury. After injury, NQO1 staining was decreased in wild type mice, but enhanced in Keap1 hypomorphs, primarily via new staining in additional tubular segments. A minor finding was the presence of rare NQO1 staining in the interstitium, suggesting fibroblast expression. Exactly how these antioxidants contribute to renal health in different cellular compartments at late stages of disease and disease recovery remains to be elucidated.

In contrast to our study, previous investigations demonstrated a protective effect of Nrf2 enhancement by both histology and function in acute IRI at the 24 hour timepoint^8^,^11^. These studies utilized pharmacologic enhancers such as bardoxolone and sulforaphane to achieve their effects, and in each case the drug had to be given as pretreatment (24 to 48 hours prior to injury) in order to be effective. In fact, in one of these studies, delaying treatment (starting treatment 3 hours prior to IRI) was ineffective in protecting against injury^8^. While Keap1 hypomorphs would be expected to simulate pretreatment, in that Nrf2 targets are already upregulated prior to injury, the differences between drug treatment and genetic enhancement are the likely cause of the observed difference between our study and previous work. In addition, the actual degree to which Nrf2 is enhanced plays an important role in the protective kinetics, and different “doses” of Nrf2 enhancement has been described to have a range of effects, ranging from protection to worsening of disease^26^,^27^. We believe that our overall findings of protection are nonetheless consistent with the majority of studies and strengthens the case for Nrf2 as a therapeutic target.

We also believe that our current work indicates a period of time in which Nrf2 enhancement can be effective in AKI. The previous studies showed that pharmacologic Nrf2 enhancement given prior to or during the AKI injury was capable of lessening kidney disease^8^,^10^. This would be helpful clinically in situations in which patients at high risk for AKI (such as CKD patients undergoing major surgery or exposure to radiological contrast material) could be pretreated to prevent injury. However, in many cases AKI is only diagnosed after injury has occurred, necessitating that therapy be initiated in a delayed fashion^41^.

Our data shows that Keap1 hypomorphism does not have an impact on the acute phase of injury (1 day post-IRI), but had profound effects in preventing chronic fibrosis 10 days after injury. Of note, ensuring equivalent severity of acute injury is one criterion proposed for studying genetically modified mice in the context of preventing AKI-to-CKD progression^41^. In this way, differential severity of the initial insult is removed as a variable contributing to CKD. With equivalence of the acute injury, our data suggests that the stimulation of Nrf2 in the subacute or “extension” phase of AKI could be helpful in preventing chronic disease. Additional studies in which pharmacologic Nrf2 enhancers are delivered in this subacute timeframe will help to test the usefulness of these agents as therapy post-AKI. This protective timeframe is reinforced by our UUO studies, in which the same initial injury is given to all mice via ureteral ligation. However, only the hypomorphs were protected from fibrosis, suggesting a role in the chronic disease process.

We must acknowledge that our study focuses on mice with genetic hypomorphism of Keap1. While Nrf2 has been widely considered to be the major target of Keap1 inhibition, it is possible that Keap1 hypomorphism has beneficial effects independent of Nrf2. We would not necessarily expect this, since experiments utilizing pharmacologic agents targeting Keap1 showed that the renoprotective effect of these agents was not present in Nrf2 null mice^8^. Nevertheless, this does not rule out effects in the specific animals used in our study.

It is noted that at 3 days post-IRI there was an improvement in serum creatinine and BUN in the Keap1 mice, whereas there was no discernible difference in the histologic injury. Certainly, although we did our best to use unbiased imaging by including the entire cortex and corticomedullary junction on a tissue section in our scoring, this outcome could still be due to sampling error. It may also be that our scoring methods were not sensitive enough to detect a difference. Finally, it may be that functional improvement preceded the change in histology, as has been described previously^42^. In any case, the serum creatinine and BUN as unbiased measures of the overall renal function should be preferred to determine overall renal function.

In conclusion, we find that Keap1 hypomorphs were protected from both IRI and UUO injury in mice, and this was associated with a reduction in inflammation and upregulation of Nrf2 target genes. The use of genetic enhancement of the Nrf2 pathway demonstrates the importance of this pathway in preventing or treating disease, and corroborates previous findings using Nrf2 enhancers. Future manipulation of this pathway will require agents that have the necessary specificity to affect this pathway with minimal off-target effects. Our results also suggest that pharmacological interventions leading to increased Nrf2 transcriptional activity may be valuable when given during the subacute extension phase of AKI, or to prevent the chronic injury after UUO. This study supports continued investigation into the protective mechanisms of the Nrf2 pathway during renal injury.

## Methods

### Animals and treatment protocol

All studies using animals were performed to the ethical and scientific standards recommended by the Guide for the Care and Use of Laboratory Animals of the NIH. The animal protocol was approved by the Institutional Animal Care and Use Committee at the University of Pittsburgh. Keap1 hypomorphs were previously described and were kindly provided by Dr. Masayuki Yamamoto^15^. Wild type animals were age- and sex-matched B6(Cg)-Tyrc-2J/J mice (catalog #000058) from The Jackson Laboratory (Bar Harbor, ME). All mice possess a C57BL/6J background. These mice were housed in the University of Pittsburgh animal facilities.

For the IRI model, mice were anesthetized, placed on a heating pad for the duration of the surgery, and the left kidney and its pedicle was isolated. The pedicle was clamped with atraumatic surgical clips (#RS-5459, Roboz, Gaithersburg, MD) for 35 minutes. The clip was then removed and reperfusion confirmed before the abdomen was closed. Mice were euthanized at 1, 3, or 10 days after reperfusion. In order to measure serum creatinine as a marker for injury, the healthy contralateral (right) kidney was removed in a second surgery exactly 24 hours prior to sacrifice. (In the case of the 1 day timepoint, the contralateral nephrectomy was done simultaneously with the IRI procedure). The healthy kidneys served as the untreated controls in all experiments. Renal tissue was divided into portions for histology and mRNA and protein isolation. Serum was also obtained.

UUO was induced as previously described^43^. Briefly, wild type and hypomorphic mice were anesthetized and the left ureter was isolated. Using 4–0 silk suture the ureter was permanently ligated and the abdomen closed. Mice were sacrificed at day 7, a timepoint at which hydronephrosis and fibrosis had developed. The healthy unobstructed kidney served as the untreated control for each mouse. Serum and tissue were collected as described above.

### Histology

Kidney tissue was fixed in 10% buffered formalin and embedded in paraffin. Three micron sections were prepared for standard histology stains. Briefly, Periodic Acid Schiff (PAS) was performed using Periodic Acid solution (#3951–100ML) and Schiff’s Reagent (#3952016–500ML) from Sigma Aldrich (St. Louis, MO) according to manufacturer’s instructions. Masson’s trichrome staining was performed with a kit (#22110648) from Thermo Fisher Scientific (Waltham, MA). Picrosirius red staining was also performed according to kit instructions (#SRS500, Scytek, Logan, UT) and slides viewed under normal light and polarized conditions.

In 1 and 3 day IRI samples, injury score was obtained by photographic evaluation of microscopic fields by an observer blinded to treatment group (R.J.T.). To avoid bias, sequential images were photographed for the entire cortex and corticomedullary junction of the section. Images were scored on the following scale: 0 = no damage; 1 = 1–25% of field showing injury; 2 = 26–50%; 3 = 51–75%; and 4 = 76–100%. Injury was defined as tubular necrosis, loss of brush borders, or tubular dilatation as previously described^10^.

Fibrosis score was obtained for both the 10 day IRI and the UUO samples. More than 10 random fields were assessed for each animal and scored in a blinded fashion according to the following system: 0 = no damage; 1 = 1–25% of field containing fibrosis; 2 = 26–50%; 3 = 51–75%; and 4 = 76–100%. In both scoring systems, numbers were averaged for each mouse and then the group average ± SEM was determined.

For NQO1 immunohistochemistry, paraffin embedded tissue was sectioned and hydrated through xylene and graded alcohols. Endogenous peroxidases were inactivated with 3% hydrogen peroxide in methanol for 15 minutes, then subjected to sequential blocking in avidin blocking solution and then biotin blocking solution for 15 minutes each (#SP-2001, Vector Laboratories, Burlingame, CA). Overnight incubation with primary antibody in 10% donkey serum was performed (#11451–1-AP, 1:100 dilution, Proteintech, Rosemont, IL). The following day the slides were washed and exposed to biotinylated secondary antibody (#711–065–152, 1:100 dilution, Jackson Immunoresearch, West Grove, PA) for 2 hours and washed. A 1 hour incubation in ABC reagent was followed by development in AEC reagent (both from Vector Laboratories) prior to coverslipping and examination.

### Biochemical measurements

Serum creatinine was determined with an assay kit from Pointe Scientific (#C7548, Canton, MI) according to included instructions. This kit utilizes an enzymatic assay which has improved sensitivity over Jaffe assays and compares favorably with HPLC methods^44^. Blood urea nitrogen (BUN) was assessed using a kit (DIUR-100) from Bioassay Systems (Hayward, CA) according to manufacturer instructions.

### Western blots

Total protein was isolated by homogenizing kidney tissue in radioimmunoprecipitation assay (RIPA) buffer supplemented with a protease inhibitor cocktail (#78442, Thermo Fisher). Homogenates were then subjected to SDS-PAGE and blotted onto PVDF membranes as previously described^45^. After blocking the blots were incubated at 4 degrees C overnight in primary antibody before exposing to horseradish peroxidase-conjugated secondary antibody and chemiluminescent substrate (Supersignal West Pico substrate, Thermo Fisher). Densitometry was performed using Image J software (NIH, Bethesda, MD) with bands normalized to an appropriate loading control.

The primary antibodies utilized were as follows: anti-fibronectin (#F3648, Sigma Aldrich), anti-α-smooth muscle actin (#A2547, Sigma Aldrich), anti-α-tubulin (#T9026, Sigma Aldrich), anti-NQO1 (#11451-1-AP, Proteintech, Rosemont, IL), anti-NGAL (AF1857, R&D Systems, Minneapolis, MN) and anti-GAPDH HRP Conjugated (#HRP-60004, Proteintech).

### Quantitative, real-time reverse transcriptase PCR (qRT-PCR)

RNA was isolated from kidney tissue homogenates using TRIzol reagent (Thermo Fisher). A reverse transcriptase reaction was performed to obtain cDNA, and this was utilized in qRT-PCR reactions using SYBR green in a CFX Connect instrument (Biorad, Hercules, CA). The primer sequences used are included (Table 1). Melt curves were utilized to ensure the specificity of the PCR product.

### Statistics

Data are presented as means ± SEM. A student’s t-test was used for comparisons between two groups. For more than two groups, one-way ANOVA with Student-Newman-Keuls or Dunn’s post tests was utilized. *P* < *0.05* was the threshold used for significance. Statistical tests were run on Sigmastat software (Systat Software, San Jose, CA).

## Additional Information

**How to cite this article**: Tan, R. J. *et al.* Keap1 Hypomorphism protects against ischemic and obstructive kidney disease. *Sci. Rep.* **6**, 36185; doi: 10.1038/srep36185 (2016).

**Publisher’s note:** Springer Nature remains neutral with regard to jurisdictional claims in published maps and institutional affiliations.

## Supplementary Material

Supplementary Information

## Figures and Tables

**Table 1:** Primers used in the qRT-PCR analyses in this study.

Gene	Forward Primer Sequence	Reverse Primer Sequence
*β-ACTIN*	CAGCTGAGAGGGAAATCGTG	CGTTGCCAATAGTGATGACC
*CATALASE*	CAATGTCACTCAGGTGCG	CAGGGTGGACGTCAGTGAAA
*GSTM1*	ATACTGGGATACTGGAACGTCC	AGTCAGGGTTGTAACAGAGCAT
*GSTP1*	GGGAGCTGCCCATACAGAC	ATGCCACCATACACCATTGTC
*IL-6*	CTTGGGACTGATGCTGGTG	CTTGGGACTGATGCTGGTG
*MCP-1*	CCCACTCACCTGCTGCTAC	TTCTTGGGGTCAGCACAGA
*MMP-7*	TAGGCGGAGATGCTCACTTT	TTCTGAATGCCTGCAATGTC
*NQO1*	AGCCAATCAGCGTTCGGTAT	GCCTCCTTCATGGCGTAGTT
*RANTES*	TGCTGCTTTGCCTACCTCTC	TTGAACCCACTTCTTCTCTGG
*α-SMA*	GAGGCACCACTGAACCCTAA	CATCTCCAGAGTCCAGCACA
*SNAIL*	ATTCTCCTGCTCCCACTGC	GACTCTTGGTGCTTGTGGAG
*SOD1*	CCAGTGCAGGACCTCATTTT	TTGTTTCTCATGGACCACCA
*SOD2*	GGCCAAGGGAGATGTTACAA	AGACACGGCTGTCAGCTTCT
*SOD3*	ATCCCACAAGCCCTAGTCT	GTGCTATGGGGACAGGAAGA
*TGF-β*	GTGGAAATCAACGGGATCAG	GTTGGTATCCAGGGCTCTCC
*TNF-α*	TCGTAGCAAACCACCAAGTG	CCTTGAAGAGAACCTGGGAG
*Keap1*	ACGTCCTCGGAGGCTATGAT	TTTGCTTCCGACAGGGTTCC
*Nrf2*	CCATTTACGGAGACCCACCGCCTG	CTCGTGTGAGATGAGCCTCTAAGCGG
*NGAL*	CCATCTATGAGCTACAAGAGAACAAT	TCTGATCCAGTAGCGACAGC
*KIM-1*	GGAATCCCATCCCATACTCCT	AAGTATGTACCTGGTGATAGCCAC
