# Supplementary material for: Keap1 hypomorphism protects against ischemic and obstructive kidney disease
Source: Sci Rep. 2016 Nov 2;6:36185. doi: 10.1038/srep36185 (PMC5090361; doi:10.1038/srep36185)
Supplement: Supplementary Information [file srep36185-s1.pdf]

Manuscript:

Keap1 hypomorphism protects against ischemia and obstructive kidney disease

Roderick J. Tan, Dionysios V. Chartoumpekis, Brittney M. Rush, Dong Zhou, Haiyan Fu, Thomas W. Kensler, and Youhua Liu

Supplemental Figure Legends

Supplemental Figure 1. Keap 1 hypomorph mice are similar to wild type mice at baseline. (**a, b**) Untreated wild type (WT) and Keap1 hypomorph (KEAP1) mice were analyzed for serum creatinine and BUN. (**c-e**) Assessment of inflammatory cytokine expression in the kidney in untreated mice. These results show that WT and KEAP1 mice have no significant baseline differences prior to injury.

Supplemental Figure 2. Keap1 hypomorphs (KEAP1) have upregulated catalase at baseline, 1 and 3 days. mRNA for catalase (**a-c**), and the three superoxide dismutases (SOD1, 2, and 3) (**d-l**) were assessed in kidneys in uninjured kidneys (control, CTL) and at 1 and 3 days after IRI. Only catalase was significantly increased in the KEAP1 mice compared to wild type mice at baseline, 1, or 3 days after IRI.

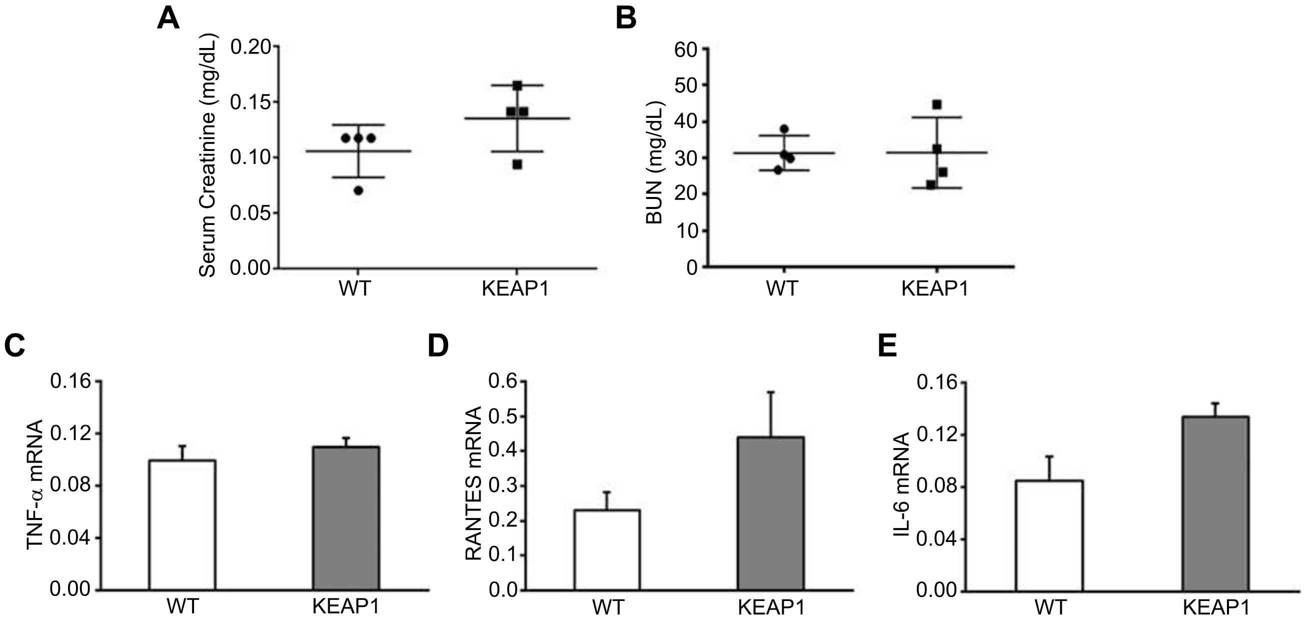

Supplemental Figure S1

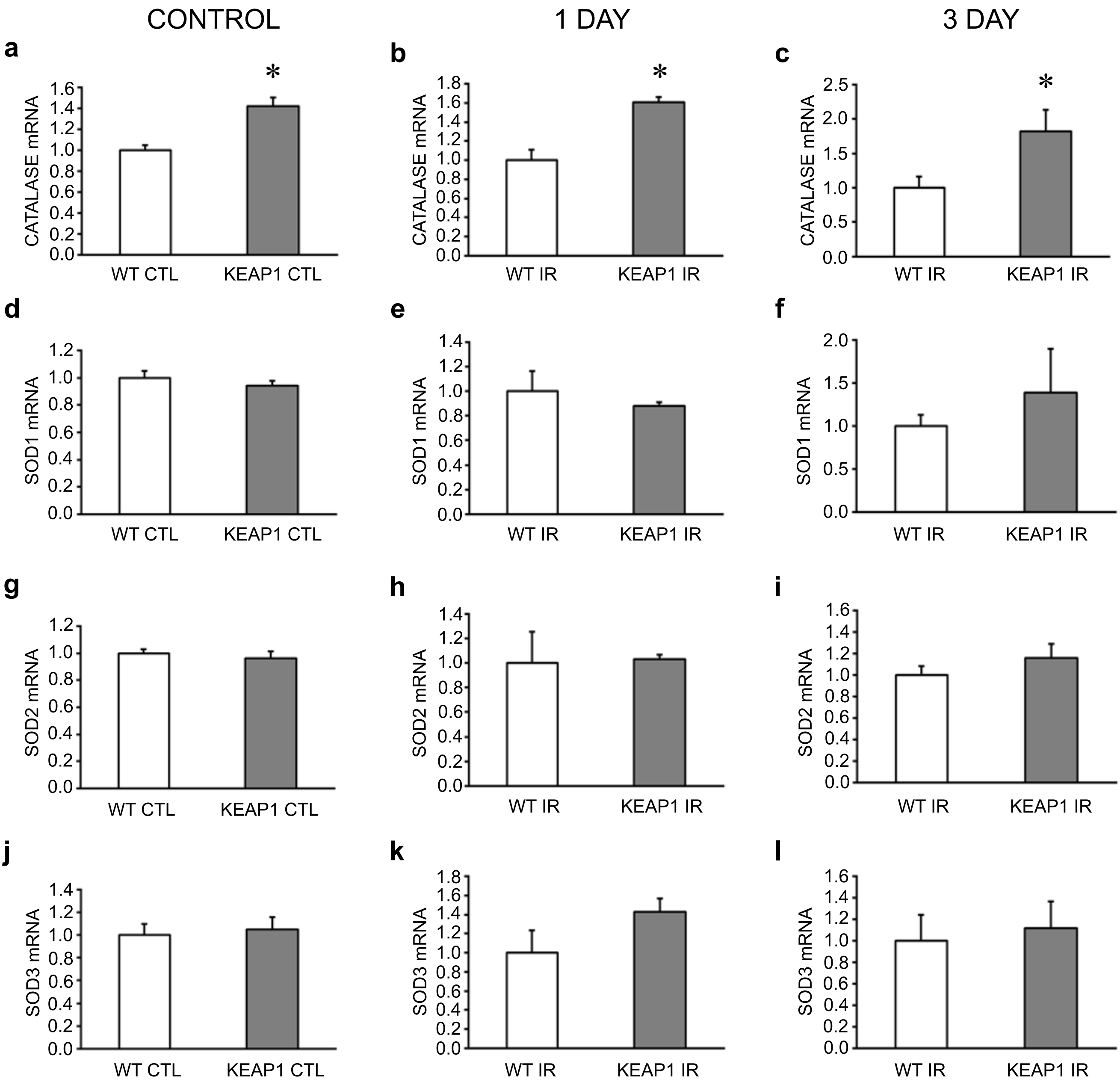

Supplemental Figure S2
